# Endothelial injury and decline in lung function in persons living with HIV: a prospective Danish cohort study including 698 adults

**DOI:** 10.3389/fmed.2024.1337609

**Published:** 2024-07-24

**Authors:** Christian Rønn, Andreas Dehlbæk Knudsen, Nicoline Stender Arentoft, Rebekka Faber Thudium, Safura-Luise Heidari, Pradeesh Sivapalan, Charlotte S. Ulrik, Thomas Benfield, Sisse Rye Ostrowski, Jens Ulrik Stæhr Jensen, Susanne D. Nielsen

**Affiliations:** ^1^Section of Respiratory Medicine, Department of Medicine, Copenhagen University Hospital – Gentofte, Hellerup, Denmark; ^2^Department of Infectious Diseases, Copenhagen University Hospital – Rigshospitalet, Copenhagen, Denmark; ^3^Department of Clinical Medicine, Faculty of Health and Medical Sciences, University of Copenhagen, Copenhagen, Denmark; ^4^Department of Respiratory Medicine, Copenhagen University Hospital - Hvidovre, Hvidovre, Denmark; ^5^Department of Infectious Diseases, Copenhagen University Hospital - Hvidovre, Hvidovre, Denmark; ^6^Department of Clinical Immunology, Copenhagen University Hospital – Rigshospitalet, Copenhagen, Denmark

**Keywords:** endothelial dysfunction, inflammation, spirometry, lung function, lung function decline, HIV

## Abstract

**Objectives:**

Endothelial injury may promote declining lung function. We aimed to investigate in well-treated persons living with HIV (PLWH) whether elevated levels of thrombomodulin (TM) and syndecan-1 (SDC1) are associated with excess lung function decline and worsening dyspnea.

**Methods:**

A prospective cohort study comprising patients from the Copenhagen municipality. We included 698 PLWH with undetectable viral load. Biomarkers and demographics were measured at baseline, spirometry [forced expiratory volume in one second (FEV_1_) and forced vital capacity (FVC)] and dyspnea score both at baseline and 2-year follow-up.

Both biomarkers were dichotomized at the 3rd quartile. Decline in lung function was estimated using a linear mixed model with patient-specific random effect. Increase in dyspnea score was estimated using a general mixed logistic regression model.

**Results:**

We did not find an association between elevated SDC1 or TM and an excess decline in neither FEV_1_: SDC1: 4.5 mL/year (95% CI: −3.9–12.9, *p* = 0.30), TM: 2.2 mL/year (95% CI: −6.0–10.4, *p* = 0.60) nor FVC: SDC1: 4.1 mL/year (95% CI: −6.0–14.2, *p* = 0.42), TM: 1.4 mL/year (95% CI: −8.3–11.1, *p* = 0.78). A subgroup analysis of never-smokers was consistent with the main analysis.

Likewise, we did not find any association between elevated SDC1 and TM and increase in dyspnea score: SDC1: OR 1.43 (95% CI: 0.89–2.30, *p* = 0.14), TM: OR 1.05 (95% CI: 0.65–1.71, *p* = 0.26).

**Conclusion:**

We did not find a significant association between elevated biomarkers of endothelial injury and decline in lung function nor dyspnea.

## Background

Persons living with HIV (PLWH), even when well-treated and without detectable viral replication, experience an increased rate of decline in lung function, as measured by forced expiratory volume in one second (FEV_1_) and forced vital capacity (FVC), compared to controls without HIV (1, 2). As a corollary, chronic obstructive pulmonary disease (COPD) is more prevalent in PLWH (3) and, low FEV_1_ in PLWH is associated with impaired quality of life and an increased risk of hospitalization (4). Likewise, low FEV_1_ in COPD is strongly associated with level of dyspnea, overall symptom burden, risk of hospitalization, and mortality (5–8).

Endothelial dysfunction is highly prevalent in all severities of COPD (9, 10), and it is well established that the pulmonary vascular endothelium is involved in chronic lung disease (11). Furthermore, it is proposed that endothelial dysfunction is not only a consequence of damaged alveolar surfaces, but rather a driving factor behind the decline in lung function and a potential target for treatment (12, 13). Additionally, PLWH exhibits increased prevalence of risk factors for endothelial dysfunction such as smoking, dyslipidemia, and abdominal obesity. Also, HIV infection has been proposed as an independent risk factor possibly through HIV associated immune activity or by direct effect on endothelial cells (14). Recent studies have found, that PLWH have elevated plasma levels of both SDC1 and TM (15, 16).

As the endothelial surface layer is being produced and shed continuously, the most used method to investigate changes in the endothelial surface layer is by measuring shed components (17, 18). Both the membrane bound expression and shedding of surface layer components may be regulated, often in response to inflammation or under conditions of cellular stress (19–21).

Syndecans are transmembrane proteins heavily involved in cellular signaling, with syndecan-1 (SDC1) exclusively found on endothelial cells (17). Elevated levels of soluble SDC1 have been associated with increased endothelial damage and worsened outcomes in COVID-19 (22), heart failure (23), pulmonary embolism (24), and renal disease (25) among others.

Thrombomodulin (TM) is a transmembrane protein involved in the antithrombogenic pathway through its binding of thrombin and activation of Protein C (26). Soluble TM has been associated with coronary heart disease and atherosclerosis (27), endothelial damage in renal disease (27), and acute respiratory distress syndrome (28).

We hypothesize that endothelial dysfunction and injury promotes excess decline in lung function in PLWH, and thus, associates with the presence of elevated shed SDC1 and TM is. Moreover, we hypothesize that elevated SDC1 and TM are associated with increased dyspnea as expressed by the medical research council (MRC) dyspnea score.

## Methods

### Study design

This was a prospective cohort study comprising patients from the Copenhagen Comorbidity in HIV infection (COCOMO) study (29). For the current study a subgroup of the COCOMO study cohort were selected based on the following inclusion criteria:

- Spirometry measured at baseline and two-year follow up.
- Age ≥ 25 years at baseline.
- HIV RNA viral load <100 copies/mL at baseline and follow up.
- CD4^+^ count >300 cells/μL at baseline and follow up.
- Initiated antiretroviral therapy ≥6 month prior to baseline visit.

Patients were followed from baseline to first follow-up after approximately 2 years. Written informed consent was signed by all patients and the COCOMO study is approved by ethics committee (approval number H-8-2014-004, ClinicalTrials.gov: NCT02382822).

### Lung function and dyspnea

Spirometry was performed to measure pre-bronchodilator values for FEV1 and FVC. Spirometry was performed two times during the study: Once at baseline and once at 2 year follow-up using a handheld EasyOne spirometer (ndd Medical, Zürich, Switzerland). Patients were instructed by staff trained in spirometry. All spirometry results were validated, as described earlier (30).

Dyspnea was assessed using the MRC score at baseline and follow-up, with questions slightly altered (Supplementary Appendix 1).

### Markers of endothelial damage

As earlier described by Ronit et al. plasma concentrations of soluble SDC1 and TM were analyzed on thawed plasma using Luminex^®^ Human Discovery Assays (R&D Systems, United Kingdom, Europe) in a 1:2 dilution, according to the manufacturer’s instructions, at the Department of Clinical Immunology, Copenhagen University Hospital, Rigshospitalet, Copenhagen, Denmark, as described earlier (29). Both biomarkers were dichotomized at the 3rd quartile in line with earlier studies (31).

### Statistical analyses

Descriptive statistics were performed on baseline data. Comparison was performed with a χ^2^ test for categorical data. For continuous data, the normally distributed data were compared with a *t* test, while non-normally distributed data were compared with Mann–Whitney Wilcoxon test.

Change in FEV1 and FVC between baseline and follow-up were expressed as mL/year to account for slight variations in follow up time. A linear mixed model was used to compare the rate of lung function decline. An adjusted model included age (continuous), sex (M/F), BMI (underweight/normal/overweight/obese), smoking status (current/former/never), and ethnicity (African/Caucasian/Hispanic/Asian/other) as fixed effects. To account for the correlation in the repeated measurements as well as possible variance over time, we included random effects (patient-specific intercept). The endothelial injury markers were added one at a time.

Risk of rapid decliner phenotype, defined by decrease in FEV_1_ ≥ 40 mL/year, according to baseline endothelial injury marker was evaluated using a general mixed logistic regression model, where the dependent variable was FEV_1_ ≥ 40 mL/year (1/0), adjusting for age (continuous), sex (M/F), BMI (underweight/normal/overweight/obese), smoking status (current/former/never), and ethnicity (African/Caucasian/Hispanic/Asian/other). The endothelial injury markers were added one at a time.

Risk of increase in dyspnea score between baseline and follow-up was evaluated using a general mixed logistic regression model, where the dependent variable was increase in MRC dyspnea score (1/0), adjusting for FEV_1_ (continuous), time (continuous), age (continuous), sex (M/F), BMI (underweight/normal/overweight/obese), smoking status (current/former/never), and ethnicity (African/Caucasian/Hispanic/Asian/other). The endothelial injury markers were added one at a time.

All analyses were first done with a crude model adjusting only for age (continuous) and sex (M/F), before applying the full adjusted model as described above.

The primary endpoint of decline in FEV_1_ was further investigated in the following sensitivity analyses: (1) stratifying for smoking status (current/former/never), (2) only using patients with airflow limitation defined by FEV_1_ < 80% of predicted and FEV_1_/FVC ratio < 0.70 at baseline, (3) including all patients with validated spirometry performed at both baseline and follow-up regardless of HIV RNA and CD4 count.

Results are presented as change in mL/year with a 95% confidence interval (CI) for change in FEV_1_ and FVC and as odds ratio (OR) with a 95% CI for risk of rapid decliner phenotype and increase in dyspnea score. *p* values <0.05 are considered significant. All statistical analyses were completed in R 4.3.0 (R Foundation for Statistical Computing, Vienna, Austria).

## Results

### Study population

**Figure 1:**
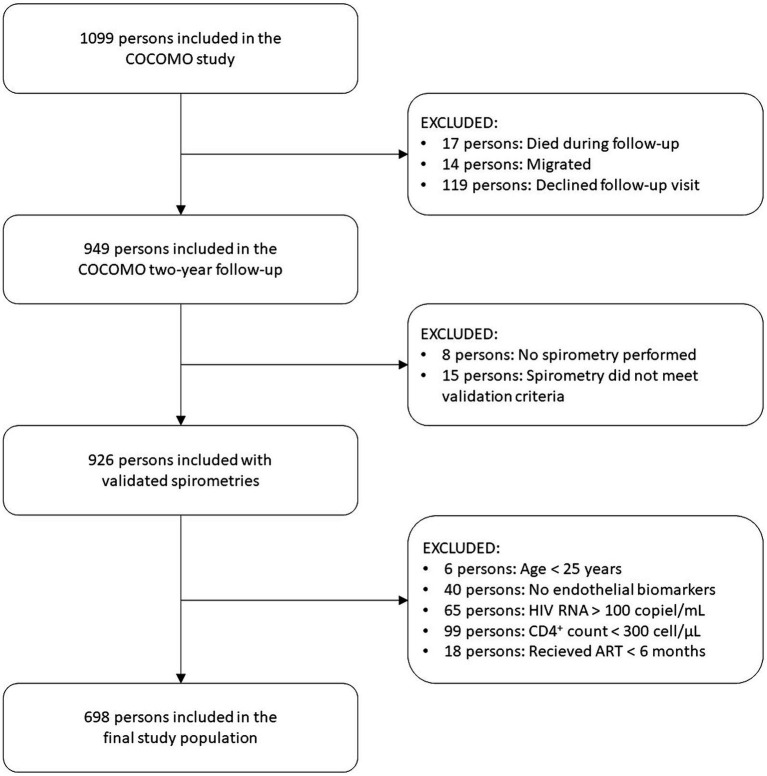
Study flow chart. ART: antiretroviral therapy.

A total of 1,099 patients were included in the COCOMO study. Of these, 926 patients had validated spirometry measured at both baseline and follow-up. Patients with missing markers of endothelial damage (*n* = 40), age < 25 years (*n* = 6), HIV RNA viral load >100 copies/mL at baseline and/or follow-up (*n* = 65), immunosuppressed with CD4 count <300 cell/μL at baseline and/or follow-up (*n* = 99), and been in antiretroviral therapy <6 months (*n* = 18) were excluded, resulting in a study population of 698 patients (see Figure 1), of whom 67 had airflow limitation.

Generally, the upper quartile group of both SDC1 and TM were in most aspects similar to the overall study population, however slightly older: Mean age upper quartile TM: 52.4 years, upper quartile SDC1: 51.5 years, overall study population: 50.3 years (for full baseline characteristics see Table 1, for graphical distribution of SDC1 and TM levels see Supplementary Appendix 2).

**Table 1:** Baseline characteristics.

Characteristic	Overall population	Syndecan above 3rd quartile		Thrombomodulin above 3rd quartile	
	(*n* = 698)	*n* = 174	*p* value	*n* = 175	*p* value
Demographics					
Age [year], median (IQR)	50.3 (43.4, 58.5)	51.5 (44.2, 61.6)	0.04	52.4 (45.4, 62.5)	<0.001
Female sex, n (%)	101 (14%)	8 (4.6%)	<0.001	19 (11%)	0.12
BMI [kg/m^2^], median (IQR)	24.4 (22.4, 27.2)	24.8 (22.0, 27.6)	0.7	24.7 (22.5, 28.0)	0.3
Ethnicity, n (%)			0.01		0.07
African	24 (3.4%)	1 (0.6%)		1 (0.6%)	
Caucasian	611 (88%)	160 (92%)		162 (93%)	
Hispanic	19 (2.7%)	3 (1.7%)		3 (1.7%)	
Asian	19 (2.7%)	1 (0.6%)		3 (1.7%)	
Other	25 (3.6%)	9 (5.2%)		6 (3.4%)	
Smoking status, n (%)			0.06		0.4
Current	183 (27%)	56 (34%)		43 (25%)	
Former	259 (38%)	62 (37%)		72 (43%)	
Never	236 (35%)	49 (29%)		54 (32%)	
Endothelial injury markers					
Syndecan-1 [ng/mL], median (IQR)	2.06 (1.79, 2.41)	2.72 (2.53, 3.05)	<0.001	2.33 (2.04, 2.76)	<0.001
Thrombomodulin [ng/mL], median (IQR)	6.59 (5.53, 7.85)	7.45 (6.29, 9.20)	<0.001	9.14 (8.33, 10.11)	<0.001
Pulmonary variables					
FEV_1_ [L], median (IQR)	3.50 (2.90, 4.03)	3.49 (2.96, 4.04)	>0.9	3.50 (2.92, 4.01)	>0.9
FEV_1_ [% of predicted], n (%)			0.14		0.7
FEV_1_ < 30%	2 (0.3%)	0 (0%)		0 (0%)	
FEV_1_ 30–49%	9 (1.3%)	5 (2.9%)		3 (1.7%)	
FEV_1_ 50–79%	135 (19%)	37 (21%)		30 (17%)	
FEV_1_ ≥ 80%	551 (79%)	132 (76%)		141 (81%)	
FVC [L], median (IQR)	4.48 (3.83, 5.14)	4.42 (3.86, 5.27)	0.8	4.54 (3.96, 5.15)	0.4
mMRC dyspnea score, n (%)			0.3		0.013
0	489 (70%)	113 (65%)		113 (65%)	
1	165 (24%)	46 (27%)		45 (26%)	
2	13 (1.9%)	5 (2.9%)		5 (2.9%)	
3	10 (1.4%)	4 (2.3%)		1 (0.6%)	
4	20 (2.9%)	5 (2.9%)		11 (6.3%)	
HIV-related variables					
CD4^+^ count [cells/μL], median (IQR)	709 (550, 890)	724 (573, 920)	0.3	730 (528, 890)	0.6
CD4^+^ nadir [cells/μL], median (IQR)	240 (130, 341)	240 (130, 370)	0.5	230 (105, 330)	0.14
HIV RNA [copies/mL], median (IQR)	19 (19, 20)	19 (19, 20)	0.6	19(19, 20)	0.7
Time with HIV [year], median (IQR)	14.4 (7.5, 22.6)	14.8 (8.6, 23.9)	0.2	15.3 (9.2, 24.3)	0.03
Inflammation markers					
IL-1β [pg/mL], median (IQR)	0.18 (0.07, 0.30)	0.19 (0.11, 0.32)	0.03	0.18 (0.07, 0.30)	>0.9
IL-10 [pg/mL], median (IQR)	0.54 (0.36, 0.82)	0.64 (0.45, 1.10)	<0.001	0.60 (0.45, 0.89)	<0.001
HS-crp [mg/L], median (IQR)	1.15 (0.57, 2.45)	1.32 (0.65, 3.21)	0.03	1.47 (0.63, 3.04)	0.01

IQR, Interquartile range; FEV_1_, Forced expiratory volume in one second; FVC, Forced vital capacity; mMRC, Modified Medical Research Council; IL-1β, Interleukin-1β; IL-10, Interleukin-10; hs-CRP, High sensitivity C-reactive protein.

### Change in FEV_1_

Overall, we found that FEV_1_ declined 38.6 mL/year (95% CI: 34.8–42.4, *p* < 0.001). An excess, though not statistically significant, decline was found in fully adjusted models for both upper quartile SDC1: 4.5 mL/year (95% CI: −3.9–12.9, *p* = 0.30) and upper quartile TM: 2.2 mL/year (95% CI: −6.0–10.4, *p* = 0.60). The adjusted results were an attenuation of the crude analyses (for crude analyses see Table 2).

**Table 2:** Change in lung function according to syndecan-1 and thrombomodulin plasma levels at baseline.

			Syndecan-1				Thrombomodulin			
			Above 3rd quartile	Below 3rd quartile	Difference	*p* value	Above 3rd quartile	Below 3rd quartile	Difference	*p* value
			β coefficient (95% CI)	β coefficient (95% CI)	(95% CI)		β coefficient (95% CI)	β coefficient (95% CI)	(95% CI)	
Change in FEV_1_ [mL/year]										
Full study population (*n* = 698)										
	Crude		−40.0 (−47.3 to −32.8)	−34.7 (−39.2 to −30.2)	−5.3 (−13.7 to 3.1)	0.23	−38.4 (−45.2 to −31.5)	−35.8 (−40.5 to −31.1)	−2.6 (−10.9 to 5.7)	0.54
	Fully adjusted		−41.7 (−48.9 to −34.5)	−37.2 (−41.8 to −32.6)	−4.5 (−12.9 to 3.9)	0.30	−40.2 (−47.0 to −33.3)	−38.0 (−42.7 to −33.3)	−2.2 (−10.4 to 6.0)	0.60
Never smokers (*n* = 236)										
	Crude		−37.8 (−50.0 to-25.5)	−23.4 (−30.7 to-16.2)	−14.3 (−28.6 to −0.1)	0.05	−36.9 (−47.4 to-26.3)	−21.3 (−29.1 to-13.4)	−15.6 (−28.7 to-2.5)	0.02
	Fully adjusted		−38.5 (−49.8 to-27.2)	−28.5 (−35.4 to-21.5)	−10.0 (−23.3 to 3.3)	0.14	−39.5 (−49.3 to-29.7)	−26.0 (−33.4 to-18.6)	−13.5 (−25.7 to −1.3)	0.03
Former smokers (*n* = 259)										
	Crude		−32.4 (−44.9 to −19.9)	−37.6 (−44.8 to −30.5)	5.2 (−9.2 to 19.6)	0.48	−35.5 (−46.6 to-24.5)	−37.2 (−44.9 to-29.5)	1.7 (−11.7 to 15.1)	0.81
	Fully adjusted		−34.6 (−47.3 to-21.9)	−38.2 (−45.2 to −31.2)	3.6 (−10.8 to 18.1)	0.63	−36.8 (−47.7 to-26.0)	−37.8 (−45.3 to −30.3)	1.0 (−12.2 to 14.1)	0.89
Current smokers (*n* = 183)										
	Crude		−49.2 (−63.3 to −35.2)	−51.5 (−62.1 to −40.8)	2.2 (−15.3 to 19.8)	0.81	−43.3 (−61.5 to-25.0)	−52.6 (−62.1 to −43.2)	9.4 (−11.1 to 29.9)	0.37
	Fully adjusted		−49.8 (−63.8 to −35.7)	−51.5 (−62.3 to −40.8)	1.7 (−15.9 to 19.4)	0.85	−43.6 (−61.9 to-25.3)	−52.7 (−62.2 to −43.2)	9.1 (−11.4 to 29.7)	0.39
Regardless of HIV RNA/CD4 count (*n* = 880)										
	Crude		−39.4 (−45.8 to −32.9)	−35.1 (−39.1 to −31.1)	−4.3 (−11.9 to 3.3)	0.26	−37.3 (−43.7 to −31.0)	−36.3 (−40.3 to −32.2)	−1.1 (−8.6 to 6.4)	0.77
	Fully adjusted		−42.3 (−48.7 to −36.0)	−38.5 (−42.5 to −34.4)	−3.9 (−11.3 to 3.5)	0.30	−40.4 (−46.7 to −34.2)	−39.4 (−43.5 to 35.4)	−1.0 (−8.4 to 6.3)	0.78
Airway limitation (*n* = 67)										
	Crude		−6.2 (−31.9 to 19.6)	−29.3 (−42.1 to −16.6)	23.2 (−5.6 to 51.9)	0.12	−13.0 (−40.0 to 14.01)	−28.6 (−40.8 to −16.5)	15.7 (−14.0 to 45.3)	0.30
	Fully adjusted		2.5 (−22.9 to 28.0)	−33.8 (−47.6 to-20.1)	36.3 (7.6 to 65.0)	0.02	−7.4 (−34.2 to 19.4)	−31.2 (−44.2 to-18.2)	23.8 (−5.4 to 53.1)	0.12
Change in FVC [mL/year]										
Full study population (*n* = 698)										
	Crude		−39.9 (−48.7 to −31.2)	−33.5 (−38.9 to-28.0)	−6.5 (−16.7 to 3.9)	0.22	−37.9 (−46.1 to-29.7)	−34.8 (−40.4 to-29.1)	−3.1 (−13.1 to 6.9)	0.54
	Fully adjusted		−41.2 (−49.8 to −32.6)	−37.1 (−42.6 to −31.6)	−4.1 (−14.2 to 6.0)	0.42	−39.6 (−47.7 to −31.4)	−38.2 (−43.8 to −32.5)	−1.4 (−11.1 to 8.3)	0.78

β, coefficient express change in lung parameter; CI, Confidence interval; FEV_1_, Forced expiratory volume in one second; FVC, Forced vital capacity.

In the sensitivity analysis stratifying for smoking status, a significant faster decline in the never smoking group (*n* = 236) was observed for both upper quartile SDC1: 14.3 mL/year (95% CI: 0.1–28.6, *p* = 0.05) and TM: 15.6 mL/year (95% CI: 2.5–28.7, *p* = 0.02). After adjustment only elevated TM remained significantly associated with a faster decline in FEV_1_: Upper quartile SDC1: 10.0 mL/year (95% CI: −3.3–23.3, *p* = 0.14) and TM: 13.5 mL/year (95% CI: 1.3–25.7, *p* = 0.03). For current and former smokers no significant results were observed (for analyses see Table 2).

In the subgroup analysis with airflow limitation (*n* = 67), upper quartile SDC1 and TM were associated with large, mostly non-significant increases in FEV_1_, probably due to random findings in a small subgroup (for analyses see Table 2).

In the sensitivity analysis including patients regardless of HIV RNA and CD4^+^ count (*n* = 880) no significant associations between upper quartile SDC1 or TM and decline in FEV_1_ were observed (for analyses see Table 2).

### Change in FVC

In general, we found that FVC declined 38.5 mL/year (95% CI: 33.8–43.2, *p* < 0.001). An excess, not significant, decline was found for both upper quartile SDC1: 4.1 mL/year (95% CI: −6.0–14.2 *p* = 0.42) and upper quartile TM: 1.4 mL/year (95% CI: −8.3–11.1, *p* = 0.78), both results fully adjusted. The adjusted results were an attenuation of the crude analyses (for crude analyses see Table 2).

### Risk for rapid decliner phenotype

We observed a significant reduction in the risk for rapid decline in FEV_1_ in the fully adjusted analysis for upper SDC1: OR 0.68 (95% CI: 0.47–0.99, *p* = 0.04) while upper quartile TM did not change risk for rapid decliner phenotype: OR: 1.04 (95% CI: 0.73–1.49, *p* = 0.83). The crude analyses were non-significant (for crude analyses see Table 3).

**Table 3:** Odds for FEV_1_ rapid decliner phenotype and increase in mMRC dyspnea score according to syndecan-1 and thrombomodulin plasma levels at baseline.

				Syndecan-1 above 3rd quartile		Thrombomodulin above 3rd quartile	
				OR (95% CI)	*p* value	OR (95% CI)	*p* value
FEV1 rapid decliner phenotype							
	Crude			0.75 (0.52 to 1.07)	0.11	1.10 (0.77 to 1.55)	0.61
	Fully adjusted				0.68 (0.47 to 0.99)	0.04	1.04 (0.73 to 1.49)	0.83
Increase in mMRC dyspnea score							
	Crude			1.62 (1.03 to 2.53)	0.03	1.14 (0.72 to 1.80)	0.49
	Fully adjusted				1.43 (0.89 to 2.30)	0.14	1.05 (0.65 to 1.71)	0.26

OR, Odds ratio; CI, Confidence interval; FEV_1_, Forced expiratory volume in one second; mMRC, Modified Medical Research Council.

### Risk for increased dyspnea score

Neither upper quartile SDC1 or TM were associated with a risk for increased dyspnea score in the fully adjusted model: SDC1: OR 1.43 (95% CI: 0.89–2.30, *p* = 0.14) and TM: OR 1.05 (95% CI: 0.65–1.71, *p* = 0.26), however in the crude model SDC1 was significantly associated with increased dyspnea score (for crude analyses see Table 3).

## Discussion

We did not find an association between the investigated biomarkers of endothelial injury, TM and SDC1, and decline in lung function or dyspnea. However, in a subgroup of never-smokers (*n* = 236), we did find a significant association between TM and SDC1 in the crude analysis with only TM remaining significant in the fully adjusted model, although not adjusting for multiple testing.

We hypothesized that endothelial dysfunction and injury might play an important role in driving the decline in lung function. Indeed, our analysis of a never-smoking population suggested that TM and SDC1 could serve as markers of endothelial injury and predict impairment in lung function. However, smoking is a very potent driver of lung function impairment and the main cause of COPD in high income countries (32). Also, smoking is a major contributor to endothelial injury and cardiovascular disease (33, 34), as is also seen in the considerably overlap of COPD and cardiovascular diseases (35). Therefore, it is possible that smoking-related endothelial damage may be not directly related to levels of SDC1 and TM.

The equilibrium between membrane bound and soluble SDC1 and TM is complex and not only influenced by endothelial injury, but also regulated by multiple factors (16, 18). As a result of this complexity, results may be difficult to interpret. A case-cohort study by Salomaa et al. observed decreasing incidence of coronary heart disease with increasing levels of soluble TM. However, the also observed an increase in carotid atherosclerosis with increasing TM (36). Similarly, a recent study by Bundgård et al. reported an association between elevated levels of soluble TM and decreased odds for peripheral arterial disease (31). The explanations may be many, but one offered by the two aforementioned studies was, that increasing levels of soluble TM reflects increasing levels of membrane bound TM which protect the endothelium and ensure an overall anticoagulant state both at and in close proximity to the endothelium and thereby reducing the risk of disease (31, 36). Interestingly, in the present study, a subgroup analysis considering only PLWH with airway limitation (*n* = 67) found that both elevated TM and SDC1 were associated with protection against decline in FEV_1_, however the subgroup was small and the findings non-significant.

PLWH with HIV RNA viral load >100 copies and/or CD4^+^ count ≤300 cells/μl were excluded from the main study, as we hypothesized that uncontrolled HIV may be a confounder both influencing SDC-1 and TM levels and lung function decline thereby distorting the association. However, the sensitivity analysis considering PLWH regardless of HIV RNA viral load and CD4^+^ count did not find any association between markers of endothelial damage and FEV_1_ decline, in line with the main analysis findings.

This study was performed on a large, well-characterized population of well-treated PLWH on antiretroviral therapy. Further strengthening the study, spirometry data underwent vigorous validation. Some limitations should be considered: Most notably the follow-up was approximately 2 years which might be too short to demonstrate a significant decline in lung function parameters associated with endothelial injury biomarkers TM and SDC1. Also, TM and SDC1 are only measured once at baseline and therefore temporal alterations in biomarker levels cannot be accounted for. Additionally, TM and SDC1 are not measured locally in the respiratory tract. In future follow-up visits it would be interesting to collect sputum samples, to establish whether biomarkers of endothelial injury in sputum is correlated to lung decline. Last, 150 patients dropped out before follow-up, which may introduce attrition bias to some degree.

To conclude, in this study comprising 698 well-treated PLWH, we did not find an association between elevated biomarkers of endothelial injury, TM and SDC1, and an accelerated decline in lung function nor an increased level of dyspnea.

## Data availability statement

The raw data supporting the conclusions of this article will be made available by the authors, without undue reservation.

## Ethics statement

This study approved by Committee on Health Research Ethics of the Capital Region of Denmark (approval number H-8-2014-004). This study was conducted in accordance with the local legislation and institutional requirements. The participants provided their written informed consent to participate in this study.

## Author contributions

CR: Conceptualization, Formal analysis, Methodology, Software, Writing – original draft. AK: Conceptualization, Methodology, Writing – review & editing. NA: Conceptualization, Methodology, Writing – review & editing. RT: Methodology, Writing – review & editing. S-LH: Methodology, Writing – review & editing. PS: Methodology, Writing – review & editing. CU: Methodology, Writing – review & editing. TB: Methodology, Writing – review & editing. SO: Methodology, Writing – review & editing. JJ: Conceptualization, Methodology, Writing – review & editing. SN: Conceptualization, Funding acquisition, Methodology, Supervision, Writing – review & editing.
