# Supplementary material for: Endothelial injury and decline in lung function in persons living with HIV: a prospective Danish cohort study including 698 adults
Source: Front Med (Lausanne). 2024 Jul 24;11:1337609. doi: 10.3389/fmed.2024.1337609 (PMC11304346; doi:10.3389/fmed.2024.1337609)
Supplement: Supplementary file 1 [file Data_Sheet_1.docx]

Supplemental Appendix 1

Grade Degree of breathlessness related to activities

0 None of the following

1 Shortness of breath when hurrying or walking uphill

2 Shortness of breath walking with peers on straight road

3 Stop for breath when walking at own pace

4 Shortness of breath when showering or when dressing
