# Supplementary material for: Endothelial injury and decline in lung function in persons living with HIV: a prospective Danish cohort study including 698 adults
Source: Front Med (Lausanne). 2024 Jul 24;11:1337609. doi: 10.3389/fmed.2024.1337609 (PMC11304346; doi:10.3389/fmed.2024.1337609)
Supplement: Supplementary file 2 [file Data_Sheet_2.docx]

Supplemental Appendix 2


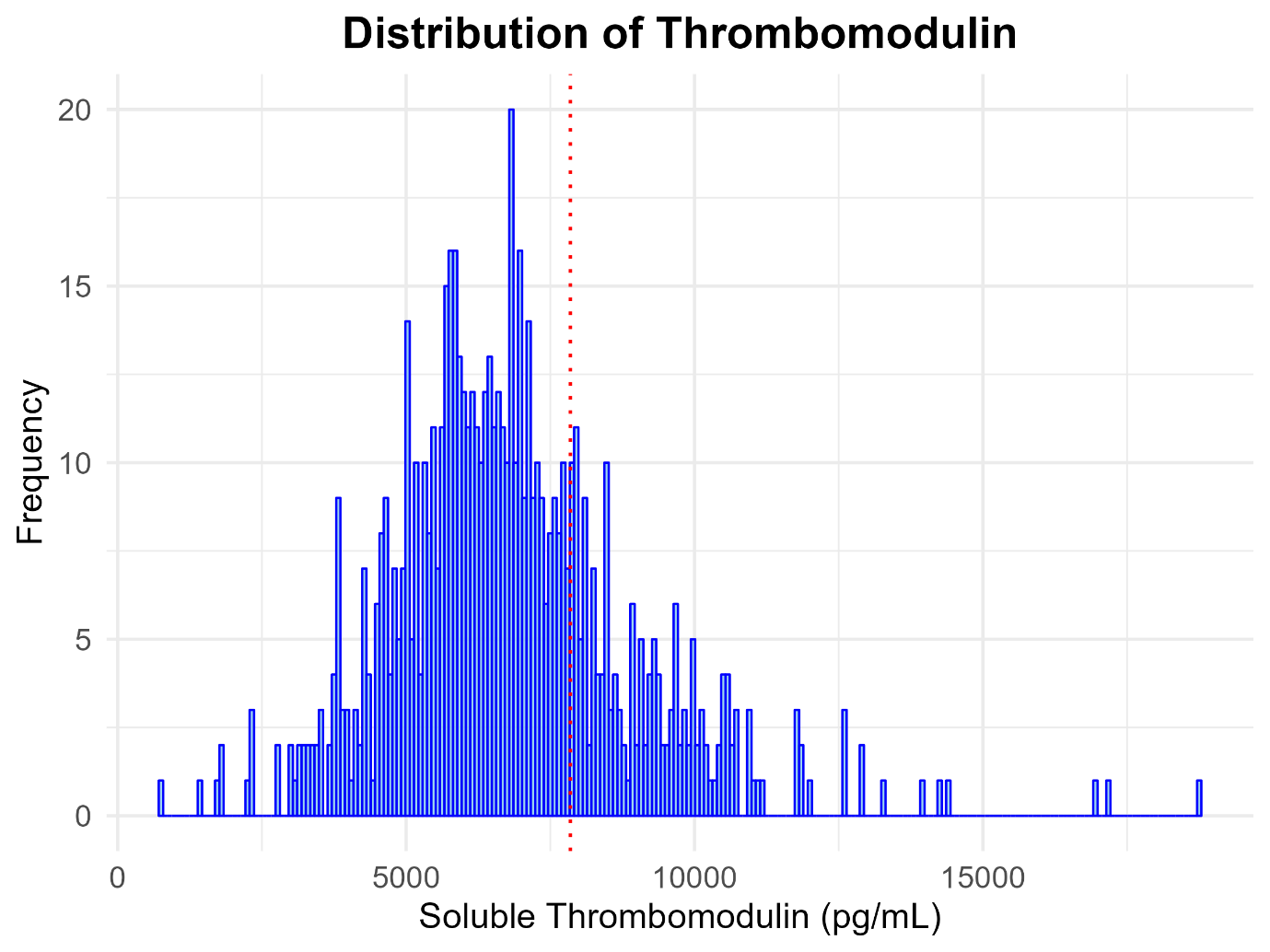


Histogram of the distribution of Trombomodulin concentrations in the study cohort. Red dotted line represents 3^rd^ quartile.


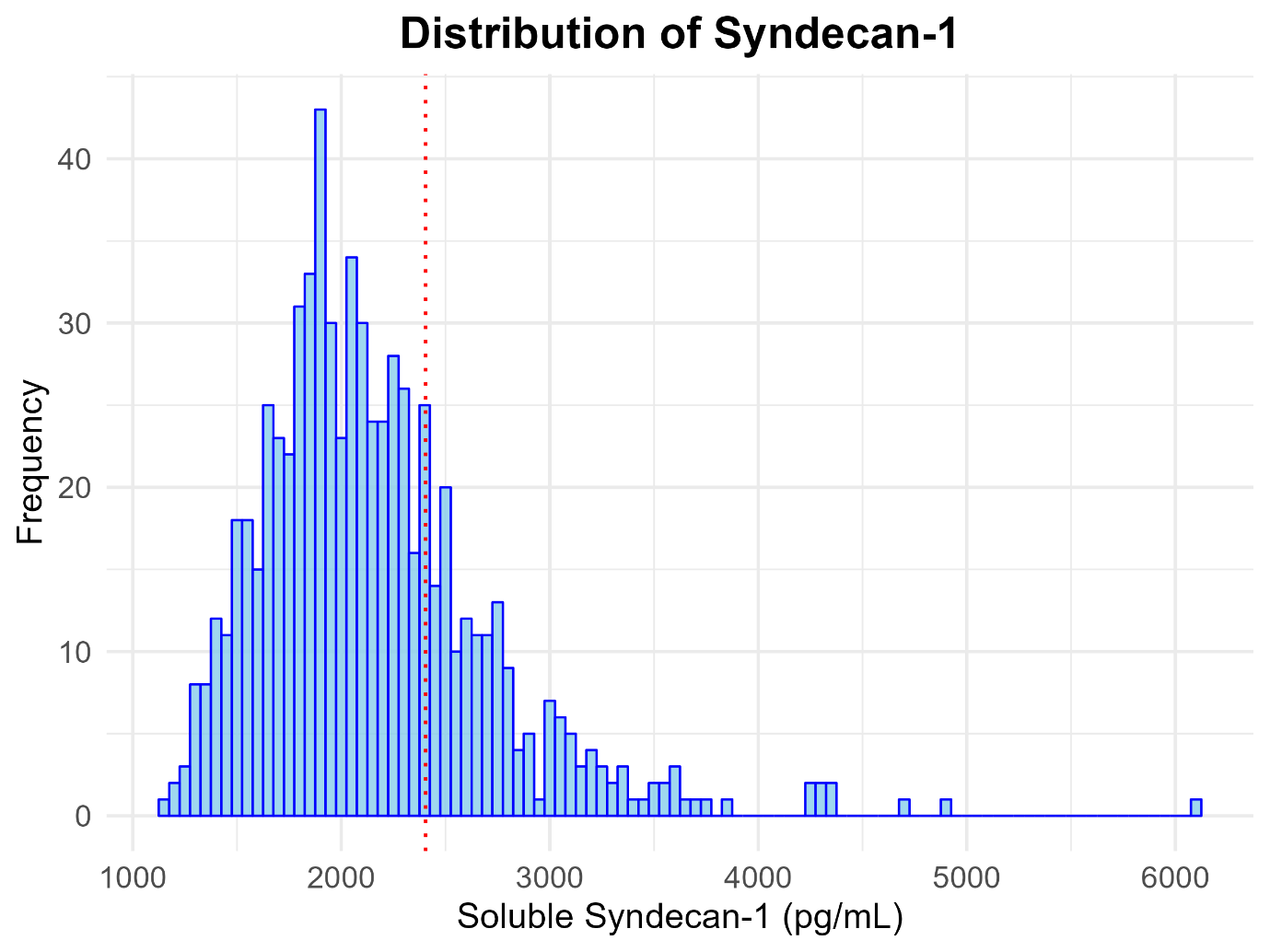


Histogram of the distribution of Syndecan-1 concentrations in the study cohort. Red dotted line represents 3^rd^ quartile.
